# COST EFFECTIVENESS ANALYSIS OF IMPLEMENTING A SCREENING PROGRAM FOR EARLY-STAGE ALZHEIMER’S DISEASE DETECTION

**DOI:** 10.1093/geroni/igae098.3570

**Published:** 2024-12-31

**Authors:** Yeongin Jung, Kaili Wu, Mashael Alnufeay, Xiaotong Jin, Jeromie Ballreich, Emmanuel Drabo

**Affiliations:** Johns Hopkins University, Baltimore, Maryland, United States; Johns Hopkins University, Baltimore, Maryland, United States; Johns Hopkins University, Baltimore, Maryland, United States; Johns Hopkins University, Baltimore, Maryland, United States; Johns Hopkins University, Baltimore, Maryland, United States; Johns Hopkins University, Baltimore, Maryland, United States

## Abstract

Recent advancements in Alzheimer’s Disease(AD) diagnosis have been propelled by the advent of disease-modifying therapies and the emergence of plasma-based biomarkers, notably P-tau217, which offers diagnostic accuracy comparable to cerebrospinal fluid assays and enables early disease detection. This study evaluates the cost-effectiveness of two AD early detection strategies for individuals aged 65 and above showing early-stage neuropathological abnormalities: solely using blood biomarker testing(P-tau217) and a sequential approach starting with plasma biomarker followed by confirmation via Aβ-PET scan. The cost-effectiveness of two early screening methods in a representative cohort of older adults in the US aged 65 and above with early-stage brain abnormalities(n=1000) is evaluated: 1) P-tau217 blood biomarker testing alone(Strategy 1); and 2) P-tau217 blood biomarker testing followed by confirmatory Aβ-PET scan(Strategy 2). Employing a hybrid model combining a decision tree and a Markov model, we simulated screening decisions, outcomes, costs, and quality-adjusted-life-years(QALYs) across AD progression stages(preclinical, mild, moderate, severe) and death over 35 years. Results suggest that Strategy 1(blood biomarker testing alone) dominates Strategy 2, producing 891 incremental QALYs at the additional cost of $49.2million translating to an incremental cost-effectiveness ratio(ICER) of $55,194/QALY gained and an incremental net monetary benefit of $39.9million at the decision threshold of $100,000/QALY gained. These results were attributable to the direct medical costs from delayed AD identification by false-negative misclassifications, and the avoided disutility from Aβ-PET. The findings were robust to uncertainties in model input parameters and highlight the utility of blood biomarkers as a cost-effective preclinical AD screening option in US older adults.

